# Protecting Neurons in Retinal and Optic Nerve Diseases: The Role of Ferroptosis-Associated Pathways

**DOI:** 10.3390/ijms27219601

**Published:** 2026-10-27

**Authors:** Lu Xiang, Xiaoyuan Ye, Amy Cheuk Yin Lo

**Affiliations:** Department of Ophthalmology, LKS Faculty of Medicine, The University of Hong Kong, Hong Kong SAR, China

**Keywords:** neuronal cell death, ischemia, cell death pathways

## Abstract

Retinal ischemia (RI) is a prominent pathological feature of several ocular diseases, including diabetic retinopathy, glaucoma, and retinopathy of prematurity. These diseases are major causes of adult and childhood blindness worldwide. Retinal and optic nerve degeneration also involves non-ischemic mechanisms. Severe or sustained RI can cause irreversible retinal injury and visual impairment. RI can trigger oxidative stress, inflammatory responses, and mitochondrial dysfunction, which can contribute to cell death. Ferroptosis is a distinct form of regulated cell death characterized by the iron-dependent accumulation of lethal lipid peroxides in membrane phospholipids. Accumulating preclinical evidence indicates that ferroptosis-associated processes may contribute to retinal and optic nerve injury. Targeting ferroptosis-related pathways may offer promising approaches to mitigate cellular damage in these diseases. In this review, we summarize the ferroptosis mechanisms relevant to retinal and optic nerve injury and evaluate evidence from experimental models, cell types, pharmacological rescue studies, and genetic approaches. By comparing experimental models, cell types, and mechanistic evidence, we distinguish direct neuronal findings from non-neuronal ocular responses and assess whether the results support ferroptosis or broader oxidative stress. We also identify the evidence needed to assess the translational relevance of ferroptosis-targeted strategies in retinal and optic nerve diseases.

## 1. Introduction

The retina, a highly metabolically active tissue essential for vision, comprises interconnected neural, glial, and vascular elements that support visual processing and retinal homeostasis. The retinal and choroidal circulations provide oxygen and nutrients required to support the metabolic demands of the resident neuronal population [1]. Impaired perfusion and oxygen delivery can cause neuronal dysfunction and injury, resulting in retinal ischemic damage, while microvascular degeneration, impaired vascular development, and pathological neovascularization contribute to conditions such as proliferative diabetic retinopathy (PDR) and retinopathy of prematurity (ROP) [2,3]. However, damage to the retina and optic nerve is not exclusively ischemic: metabolic disturbances, pressure-related stress, and direct axonal injury also contribute to degeneration in the human diseases and experimental models considered in this review. Extensive research into the underlying molecular mechanisms has revealed the involvement of retinal cellular dysfunction and the activation of multiple overlapping injury pathways [4,5,6]. In particular, ferroptosis-associated mechanisms have emerged as potential contributors to retinal and optic nerve injury.

Ferroptosis is an iron-dependent form of regulated cell death (RCD) marked by the lethal accumulation of lipid peroxides in membrane phospholipids (PLs) [7]. Its regulation encompasses iron homeostasis, lipid metabolism, and redox processes, collectively orchestrated by numerous enzymatic reactions and molecular interactions across cell compartments, including the cell membrane, cytoplasm, and various organelles [8,9]. Ferroptosis occurs when the generation and propagation of lipid peroxides in membrane PLs exceed cellular lipid-peroxide detoxification capacity. PLs containing acyl chains derived from polyunsaturated fatty acids (PUFAs) are particularly susceptible to lipid peroxidation. Their oxidation can occur through both enzymatic (mediated by iron-dependent enzymes such as lipoxygenases, LOXs) and nonenzymatic (facilitated by redox-active metals, particularly iron) processes in a context-dependent manner, ultimately producing phospholipid hydroperoxides (PLOOHs) [10].

Cellular susceptibility to ferroptosis therefore depends, in part, on the capacity to limit lipid peroxidation and detoxify PLOOHs [7,8,9,10]. Lipid peroxide detoxification is primarily facilitated by the canonical reduced glutathione (GSH)/glutathione peroxidase 4 (GPX4) pathway. Using GSH as a reducing substrate, GPX4 converts PLOOHs into corresponding phospholipid alcohols (PLOHs) [7,8,11,12]. Beyond GPX4, ferroptosis defenses involve reduced coenzyme Q (ubiquinol, CoQH_2_) regeneration associated with ferroptosis suppressor protein 1 (FSP1) and dihydroorotate dehydrogenase (DHODH), as well as antioxidant activity and lipid remodeling linked to the guanosine triphosphate (GTP) cyclohydrolase 1 (GCH1)/tetrahydrobiopterin (BH_4_) pathway [13,14,15]. Therefore, investigating key molecular components of the ferroptosis pathway in specific retinal cell types may offer mechanistic insight into the pathogenesis of retinal and optic nerve injury.

Importantly, changes in individual oxidative stress markers or ferroptosis-regulator expression alone do not establish ferroptotic cell death. Increased levels of reactive oxygen species (ROS), malondialdehyde (MDA), 4-hydroxynonenal, or total iron, as well as altered expression of GPX4, solute carrier family 7 member 11 (SLC7A11), or acyl-CoA synthetase long-chain family member 4 (ACSL4), can accompany broader oxidative injury. Stronger attribution requires converging evidence linking cell death to iron-dependent membrane lipid peroxidation, supported by appropriate pharmacological rescue and, where feasible, genetic perturbation of relevant regulators. No single marker or rescue intervention is definitive [7,8,9]. Accordingly, throughout this review, we describe isolated molecular alterations as ferroptosis-associated changes or findings consistent with ferroptotic stress, reserving attribution to ferroptotic cell death for findings supported by convergent functional and mechanistic evidence. Here, we outline the mechanisms relevant to ferroptosis in the retina, appraise available ocular evidence across disease models and cell types, and discuss the implications and limitations of ferroptosis-targeted approaches for retinal and optic nerve diseases.

The review complements existing summaries of ferroptosis-related pathways by comparing the experimental models used, the cell types studied, and the mechanistic evidence obtained. Comparisons across these dimensions help distinguish findings in acute injury models from those supported by chronic disease models, as well as direct neuronal evidence from non-neuronal ocular responses. Evaluating marker changes alongside pharmacological rescue and genetic perturbation helps assess the strength of evidence supporting ferroptotic cell death. Together, this review clarifies the applicability of the reported mechanisms and identifies the limitations of the current evidence without proposing a new ferroptosis mechanism.

## 2. Mechanisms of Ferroptosis

### 2.1. Iron Homeostasis and Ferroptosis Susceptibility

#### 2.1.1. Iron Homeostasis

**Figure 1:**
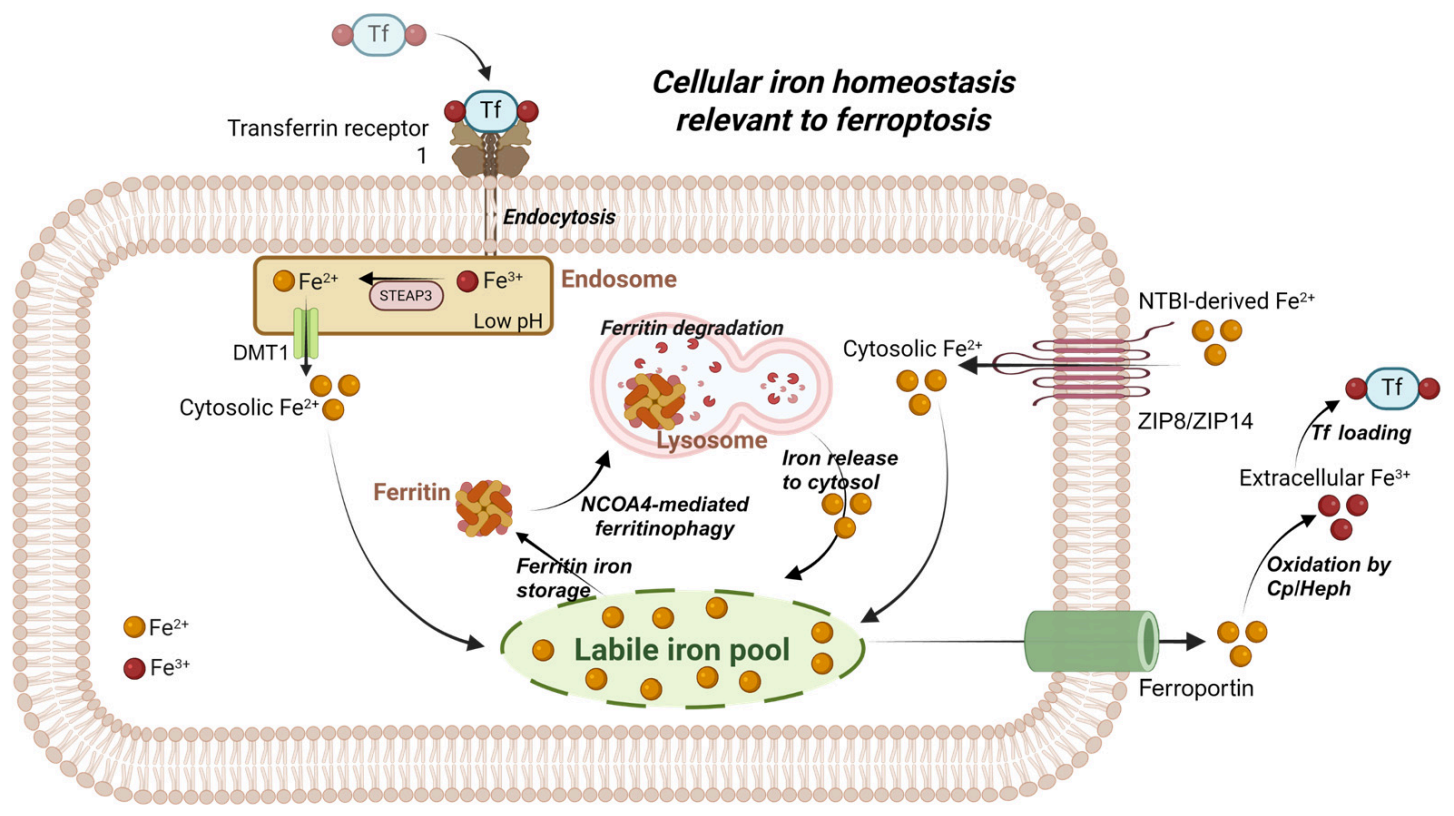
Cellular iron-homeostasis pathways relevant to ferroptosis. Following TfR1-mediated endocytosis, endosomal acidification promotes the release of Fe^3+^ from Tf. In the canonical uptake pathway, STEAP3 reduces Fe^3+^ to Fe^2+^, which DMT1 transports across the endosomal membrane into the cytosol. Cytosolic iron contributes to the LIP or is sequestered in the iron-storage protein ferritin. NCOA4 mediates the delivery of ferritin for lysosomal degradation through ferritinophagy, releasing iron that can subsequently enter the cytosolic LIP. Ferroportin exports Fe^2+^, while Cp and Heph oxidize extracellular Fe^2+^ to Fe^3+^, facilitating Tf loading. ZIP8 and ZIP14 can mediate NTBI uptake; their expression and regulation have been demonstrated in the mouse retina, while their cell-specific transport contributions require further investigation. The schematic diagram illustrates selected canonical mechanisms established in different experimental systems and does not imply direct validation of every pathway in retinal cells. Abbreviations: Cp, ceruloplasmin; DMT1, divalent metal transporter 1; Fe^2+^, ferrous iron; Fe^3+^, ferric iron; Heph, hephaestin; LIP, labile iron pool; NCOA4, nuclear receptor coactivator 4; NTBI, non-transferrin-bound iron; STEAP3, six-transmembrane epithelial antigen of the prostate 3; Tf, transferrin; TfR1, transferrin receptor 1; ZIP8/ZIP14, Zrt- and Irt-like proteins 8 and 14. Created in BioRender. Xiang, L. (2026) https://BioRender.com/kz2tz2r.

The retina is composed of the neural retina and the retinal pigment epithelium (RPE). The neural retina is organized into distinct layers, including the nerve fiber layer, ganglion cell layer (GCL), inner plexiform layer, inner nuclear layer, outer plexiform layer, outer nuclear layer, and photoreceptor layers [16]. Retinal cells can acquire iron through two principal routes: transferrin-bound iron (TBI) uptake and non-transferrin-bound iron (NTBI) uptake (Figure 1). TBI refers to ferric iron (Fe^3+^) bound to transferrin (Tf) for cellular uptake via the transferrin receptor (TfR) on the cell surface [17]. TfR expression has been detected in the GCL, inner nuclear layer, outer plexiform layer, photoreceptor inner segments, and RPE [18,19]. TfRs on both apical and basolateral surfaces of cultured human RPE participate in receptor-mediated endocytosis. Basolateral uptake provides a potential route for acquiring TBI from the choroidal circulation [20]. Beyond canonical Tf-TfR-mediated endocytosis, Zrt- and Irt-like proteins ZIP8 and ZIP14 are expressed in the mouse retina and are regulated in experimental retinal iron overload, supporting their potential involvement in NTBI uptake [21]. Müller cells span the neural retina and may contribute to retinal iron homeostasis; however, their specific roles require further investigation.

After cellular uptake in the Tf-dependent pathway, the reduced pH inside the endosome promotes Fe^3+^ dissociation from the Tf-TfR complex [22]. In the canonical Tf uptake pathway, six-transmembrane epithelial antigen of the prostate 3 (STEAP3) converts Fe^3+^ to ferrous iron (Fe^2+^) in erythroid cells and is a candidate metalloreductase for iron handling in other cell types, although its retinal role remains incompletely defined [23]. Fe^2+^ is then exported from endosomes into the cytosol through divalent metal transporter 1 (DMT1) on the endosomal membrane [24]. Notably, DMT1, a proton/Fe^2+^ symporter, is expressed in photoreceptors as well as horizontal and bipolar cells, consistent with a role in retinal iron utilization [25]. Following iron release, the Tf-TfR complex returns to the cell membrane. At near-neutral extracellular pH, iron-free Tf dissociates from TfR and can subsequently bind iron and re-enter the uptake cycle [26].

Within the cytoplasm, iron enters the labile iron pool (LIP), from which it can be utilized for cellular functions or sequestered in ferritin (Ft). The LIP is a metabolically accessible pool of loosely bound iron that can be used in various cellular processes [27]. Cytosolic Ft is a major intracellular iron-storage protein comprising 24 subunits in variable proportions of ferritin heavy chain (FTH) and ferritin light chain (FTL). FTH provides ferroxidase activity, oxidizing Fe^2+^ to Fe^3+^ for storage within the ferritin core, whereas FTL contributes to ferritin stability and long-term iron storage [28]. Ft is expressed in all retinal layers, prominently in the GCL, inner nuclear layer, outer plexiform layer, inner segments of photoreceptors, and RPE [18]. During lysosomal degradation of Ft, including ferritinophagy, released iron can enter the LIP for subsequent cellular utilization [29].

Iron export is mediated by ferroportin (Fpn), the sole known iron export protein [30], which exports Fe^2+^. Ferroxidases such as ceruloplasmin (Cp) and hephaestin (Heph) oxidize exported Fe^2+^ to Fe^3+^, thereby facilitating extracellular iron handling and iron loading onto Tf [31]. In the retina, these enzymes are co-expressed with Fpn in RPE and Müller cells and facilitate iron efflux [31,32]. Proposed routes of iron movement out of retinal cells include basolateral Fpn-mediated export from the RPE toward the choroidal circulation and export at Müller-cell end-feet toward the vitreous [25,32]. In addition to Fpn-mediated iron export, iron-rich Ft can be secreted through a multivesicular body-exosome pathway [33]. In mammary epithelial and breast carcinoma cells, prominin2-dependent formation of Ft-containing multivesicular bodies and exosomes limited intracellular iron accumulation and promoted ferroptosis resistance [34]. These findings identify an alternative iron-export mechanism in non-retinal systems; its contribution to retinal iron homeostasis and ferroptosis susceptibility requires further investigation.

#### 2.1.2. Iron Regulation

Retinal iron homeostasis depends on coordinated regulation of iron uptake, storage, and export through TfR, Ft, and Fpn, and their associated regulatory pathways [18,31,32,35]. TfR has two major subtypes, transferrin receptor 1 (TfR1) and transferrin receptor 2 (TfR2), which have distinct roles in iron uptake and regulation. In the retina, the hemochromatosis-associated protein HFE, hepcidin (Hepc), and Fpn exhibit cell-type-specific expression [19]. HFE, a major histocompatibility complex class I-like protein, is localized to the basolateral membrane of RPE cells, consistent with a potential role in local iron sensing and regulation [19,35].

Mechanistic studies in non-retinal systems have shown that HFE interacts with both TfR1 and TfR2 with distinct regulatory effects [36,37]. HFE and Fe^3+^-Tf compete for overlapping binding sites on TfR1, providing a mechanism through which HFE can influence TfR1-mediated iron uptake [37]. In systemic iron homeostasis, TfR2 and HFE participate in the regulation of Hepc expression, whereas Hepc limits Fpn-mediated iron export by promoting Fpn internalization and degradation [36,38].

In the mouse retina, Hepc expression has been detected in Müller cells, photoreceptors, and RPE cells. Lipopolysaccharide (LPS)-induced Hepc upregulation was associated with reduced Fpn abundance, supporting a potential role for locally produced Hepc in retinal iron regulation. The study implicated Toll-like receptor 4 (TLR4) in the response to LPS and demonstrated that LPS-induced Hepc upregulation persisted in primary RPE cultures from *Hfe-null* mice [39]. However, the extent to which the full HFE-TfR2-Hepc regulatory axis operates in individual retinal cell types requires further investigation.

### 2.2. Lipid Peroxide Accumulation

#### 2.2.1. Lipid Metabolism in the Retina

Retinal neurons have high metabolic demands and rely on glucose for their energy supply. Lipid metabolism also supports membrane synthesis, lipid remodeling, and signaling, while fatty-acid oxidation contributes to retinal energy homeostasis and cellular function [40,41,42]. Retinal lipid metabolism is regulated in part by factors such as the nuclear receptor PPARα (peroxisome proliferator-activated receptor α), which governs expression of genes involved in lipid uptake, transport, and oxidation [43]. Disruptions or imbalances in retinal lipid metabolism are closely associated with several degenerative retinal and optic-nerve disorders, underscoring the critical role of lipid homeostasis in preserving retinal integrity and function [44].

**Figure 2:**
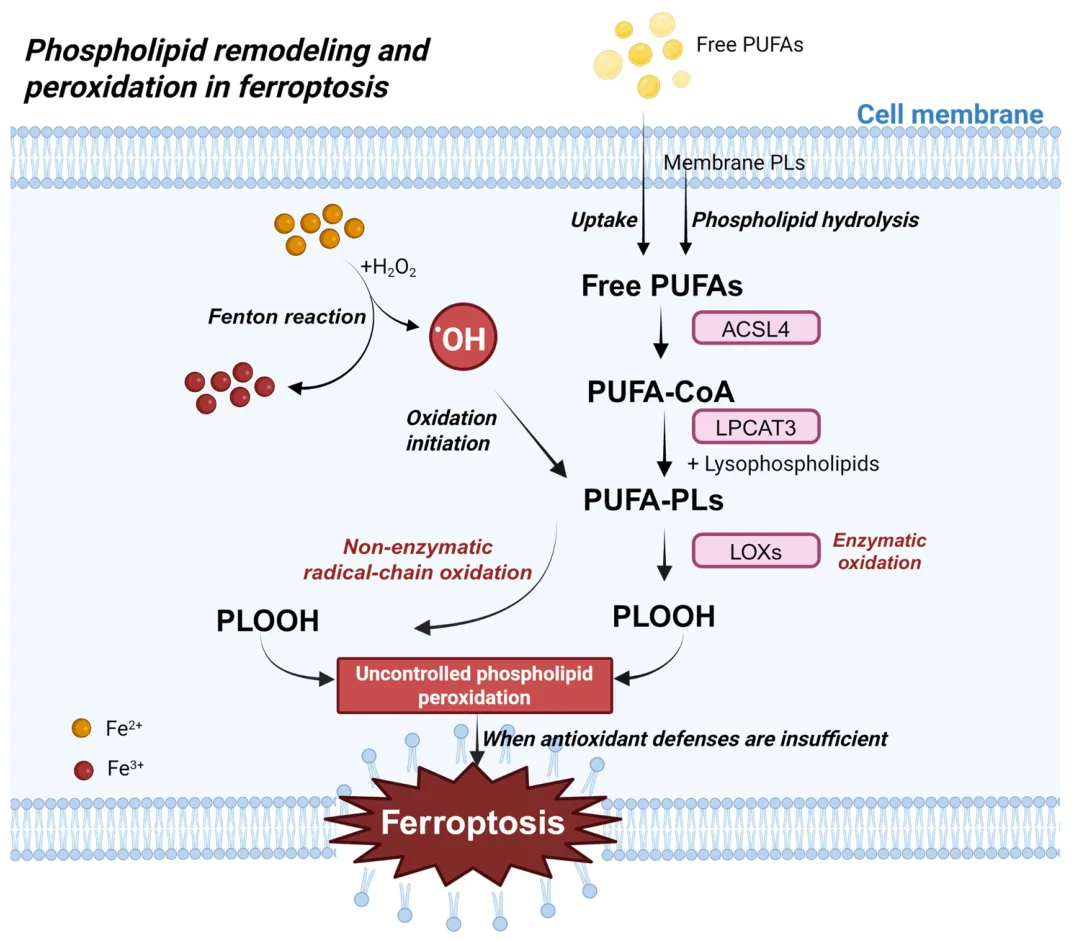
Phospholipid remodeling and lipid peroxidation relevant to ferroptosis. Free PUFAs obtained through cellular uptake or phospholipid hydrolysis are converted by ACSL4 into their corresponding acyl-CoAs. LPCAT3 transfers fatty acyl groups to lysophospholipids, generating PUFA-PLs. The resulting PUFA-PLs are susceptible to enzymatic or non-enzymatic oxidation. In the simplified Fenton reaction scheme, Fe^2+^ reacts with H_2_O_2_ to generate Fe^3+^ and hydroxyl radicals, which can initiate phospholipid oxidation. Uncontrolled phospholipid peroxidation can promote membrane damage and ferroptosis when cellular antioxidant defenses are insufficient. The schematic diagram illustrates selected mechanisms rather than a universally required sequence. Abbreviations: ACSL4, acyl-CoA synthetase long-chain family member 4; Fe^2+^, ferrous iron; Fe^3+^, ferric iron; H_2_O_2_, hydrogen peroxide; LOXs, lipoxygenases; LPCAT3, lysophosphatidylcholine acyltransferase 3; PLs, phospholipids; PLOOH, phospholipid hydroperoxide; PUFAs, polyunsaturated fatty acids; PUFA-CoA, polyunsaturated fatty acyl-coenzyme A; PUFA-PLs, polyunsaturated fatty acid-containing phospholipids; •OH, hydroxyl radical. Created in BioRender. Xiang, L. (2026) https://BioRender.com/f6c258u.

Retinal membranes are enriched in omega-3 and omega-6 long-chain polyunsaturated fatty acids (LC-PUFAs) [45]. These LC-PUFAs, particularly docosahexaenoic acid (DHA), are crucial for maintaining the structural integrity of photoreceptor membranes and supporting visual transduction [45,46]. This fatty acid composition enhances membrane fluidity, a vital factor for proper signal transmission and photoreceptor health [45]. However, the multiple carbon–carbon double bonds in PUFAs weaken adjacent C-H bonds, increasing PUFA susceptibility to free-radical attack and subsequent lipid peroxidation (Figure 2) [47].

#### 2.2.2. Modulation of Lipid Peroxidation

Lipid peroxidation, a central process in ferroptosis, involves initiation, propagation, and termination of lipid–radical reactions. Initiation can occur through iron-dependent radical chemistry, while enzymatic processes can also contribute to lipid oxidation [10]. In Fenton chemistry, Fe^2+^ reacts with H_2_O_2_ to generate hydroxyl radicals, which can remove hydrogen atoms from PUFA chains and initiate lipid radical formation. Enzymes can also promote lipid peroxidation indirectly. Oxidoreductases, including cytochrome P450 oxidoreductase (POR) and cytochrome b5 reductase 1 (CYB5R1), have been shown to generate H_2_O_2_, which supports iron-dependent radical formation and subsequent PL oxidation [48]. LOXs can contribute to PUFA oxidation and lipid-hydroperoxide formation, although their roles in ferroptosis vary with cell type and experimental context [49,50]. During propagation, lipid radicals react with molecular oxygen to form lipid peroxyl radicals that extract hydrogen from neighboring lipids, producing hydroperoxides and new lipid radicals. Lipid hydroperoxides accumulate as major products of these reactions, which can promote membrane damage when their formation exceeds detoxification capacity.

The key substrates of ferroptotic lipid peroxidation are polyunsaturated fatty acid-containing phospholipids (PUFA-PLs) rather than free PUFAs. The abundance and composition of PUFA-PLs are influenced by enzymes involved in fatty-acid activation and PL remodeling [51,52,53]. In one well-characterized pathway, ACSL4 activates selected PUFAs, including arachidonic acid and adrenic acid, by converting them into their corresponding acyl-CoA derivatives [51]. Lysophosphatidylcholine acyltransferase 3 (LPCAT3) subsequently transfers fatty acyl groups from these acyl-CoAs to lysophospholipids, contributing to the formation of arachidonoyl- and adrenoyl-containing phosphatidylethanolamines [53]. In non-retinal cancer models, phosphoinositide 3-kinase (PI3K)/protein kinase B (AKT)/mechanistic target of rapamycin complex 1 (mTORC1) signaling suppressed ferroptosis. mTORC1 activated sterol regulatory element-binding protein 1 (SREBP1) and thus enhanced the transcription of stearoyl-CoA desaturase-1 (SCD1) [54], which promoted the synthesis of monounsaturated fatty acids (MUFAs). Unlike PUFAs, MUFAs lack bis-allylic hydrogen atoms and are less susceptible to lipid peroxidation. Their incorporation into membrane PLs can decrease the abundance of oxidizable PUFA-containing species, thereby reducing susceptibility to ferroptosis [55,56].

In the outer retina, PUFA-rich photoreceptor membranes, high oxygen availability, and chronic light exposure create a substantial oxidative challenge [57,58]. Continuous photoreceptor outer-segment renewal also requires extensive lipid processing and recycling by photoreceptors and RPE cells [57]. These cells rely on regulated iron handling and antioxidant defenses to limit lipid peroxide accumulation [57,58]. Experimental retinal models showed that ferrostatin-1 (Fer-1) attenuated light-induced retinal degeneration and reduced pathological angiogenesis in oxygen-induced retinopathy (OIR), supporting a pathogenic role of uncontrolled lipid peroxidation in retinal injury [59,60].

Retinal cells possess antioxidant defenses, including the GSH/GPX4 axis, that help limit lipid peroxidation and support cell survival [7,47,61]. When lipid-peroxide generation exceeds cellular detoxification capacity, oxidative membrane damage can accumulate and contribute to cell injury. However, attribution to ferroptosis requires complementary functional and mechanistic evidence [7,8,9,10].

### 2.3. Antioxidant Defense

Antioxidants can limit lipid peroxidation by preventing its initiation or trapping chain-propagating radicals [62]. Radical-trapping antioxidants (RTAs) interrupt lipid-peroxidation chain reactions and thereby counteract a central process underlying ferroptosis [7,62]. Such protection is particularly relevant to retinal neurons, including photoreceptors and retinal ganglion cells (RGCs), because of their high metabolic demand and susceptibility to lipid-peroxidation injury [40,57,59,63].

**Figure 3:**
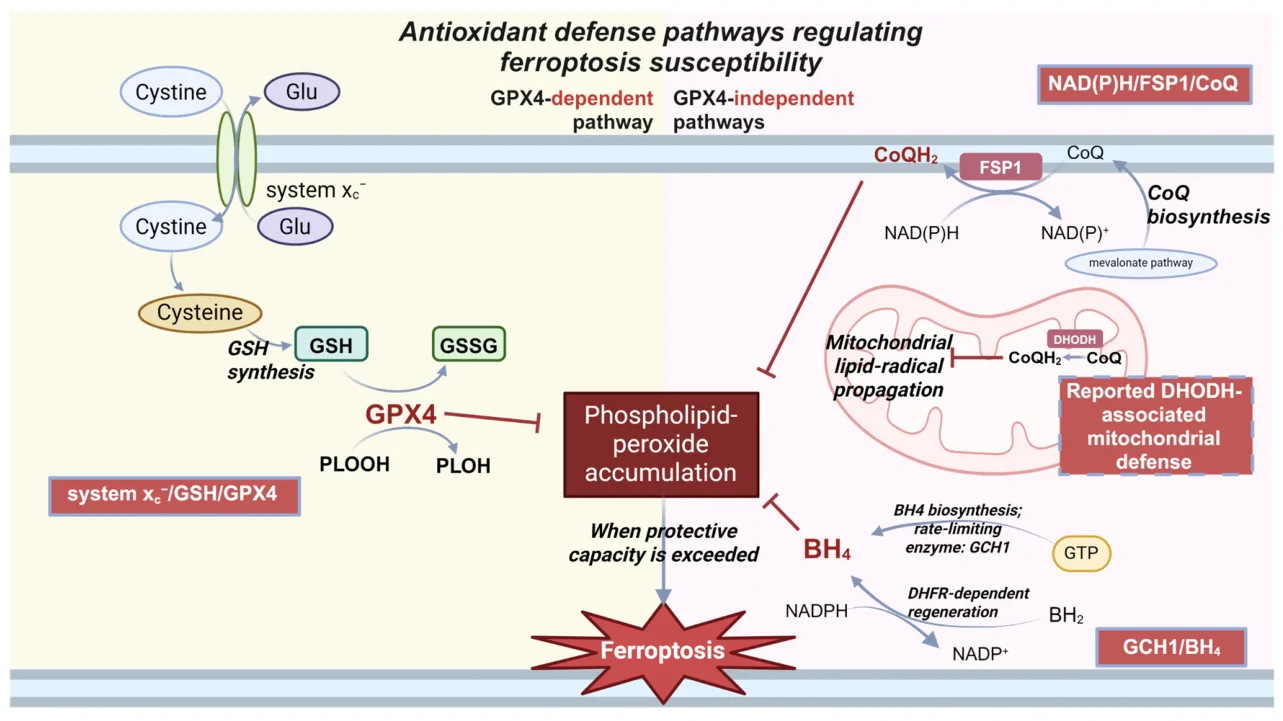
Antioxidant defense pathways regulating ferroptosis susceptibility. System x_c_^−^ imports cystine in exchange for glutamate. Intracellular cystine is reduced to cysteine, which supports GSH synthesis. GPX4 uses GSH to reduce PLOOH to PLOH, producing GSSG. Independently of GPX4, FSP1 uses NAD(P)H to regenerate CoQH_2_ from CoQ, limiting lipid-radical propagation. The mevalonate pathway contributes to CoQ biosynthesis. GCH1 initiates BH_4_ biosynthesis, while DHFR regenerates BH_4_ from BH_2_ using NADPH. BH_4_ contributes radical-trapping antioxidant protection. At the mitochondrial inner membrane, DHODH couples dihydroorotate oxidation to CoQ reduction and has been reported to suppress mitochondrial lipid peroxidation. The dashed outline identifies this context-dependent defense pathway, for which inhibitor specificity requires consideration. Together, these defenses limit phospholipid-peroxide accumulation; ferroptotic death can occur when protective capacity is exceeded. The schematic diagram combines findings from different experimental systems and does not imply direct validation of every pathway in retinal cells. Blue arrows indicate biochemical pathways, whereas red T-shaped lines indicate inhibitory effects on phospholipid-peroxide accumulation or lipid-radical propagation. Abbreviations: BH_2_, dihydrobiopterin; BH_4_, tetrahydrobiopterin; CoQ, coenzyme Q (ubiquinone); CoQH_2_, reduced coenzyme Q (ubiquinol); DHFR, dihydrofolate reductase; DHODH, dihydroorotate dehydrogenase; FSP1, ferroptosis suppressor protein 1; GCH1, GTP cyclohydrolase 1; Glu, glutamate; GPX4, glutathione peroxidase 4; GSH, reduced glutathione; GSSG, glutathione disulfide; GTP, guanosine triphosphate; NAD(P)^+^, oxidized nicotinamide adenine dinucleotide (phosphate); NAD(P)H, reduced nicotinamide adenine dinucleotide (phosphate); PLOH, phospholipid alcohol; PLOOH, phospholipid hydroperoxide; system x_c_^−^, cystine-glutamate antiporter. Created in BioRender. Xiang, L. (2026) https://BioRender.com/shp7e90.

Cellular defenses against ferroptosis comprise GPX4-dependent and GPX4-independent pathways (Figure 3). GPX4 reduces PLOOHs to their corresponding PLOHs, thereby limiting PL peroxide accumulation [7,12]. GPX4-independent defenses include FSP1-associated regeneration of CoQH_2_ and GCH1/BH_4_-associated antioxidant activity and lipid remodeling [13,15]. DHODH-associated CoQH_2_ regeneration has also been reported to suppress mitochondrial lipid peroxidation, although its contribution is context-dependent [14,64]. In addition, the PL-remodeling enzymes membrane-bound O-acyltransferase domain-containing 1 and 2 (MBOAT1/2) suppress ferroptosis by incorporating MUFAs into membrane PLs in non-retinal experimental systems [65]. The following sections discuss these pathways while distinguishing direct retinal evidence from mechanisms established in other cellular systems.

#### 2.3.1. Glutathione Peroxidase 4-Dependent Pathway

GPX4 is an antioxidant enzyme highly dependent on selenium. It is one of the eight phylogenetically related glutathione peroxidases (GPX1-8) involved in cellular antioxidant defense [12]. GPX4 is distinguished by its ability to directly reduce PL and cholesterol hydroperoxides in membranes and lipoproteins, thereby protecting cells against lipid-peroxidation-driven injury [12,66]. Three isoforms of GPX4 have been described: cytosolic GPX4, mitochondrial GPX4, and sperm-nuclear GPX4 [66]. Global GPX4 depletion causes embryonic lethality in mice, whereas inducible deletion in adult mice produces a lethal phenotype accompanied by neuronal loss [66,67,68].

The cystine-glutamate antiporter (system x_c_^−^) imports cystine in exchange for glutamate [7,8]. The system x_c_^−^/GSH/GPX4 axis is a central ferroptosis-defense system [7,8]. Intracellular cystine is subsequently reduced to cysteine, which, together with glutamate and glycine, forms the tripeptide GSH [7,8]. GSH is the primary reducing substrate for GPX4 and plays a crucial role in cellular antioxidant defense [7,12]. In the retina, impaired antioxidant defenses, including changes in GSH/GPX4, have been reported in experimental RGC and photoreceptor injury [59,63]. Ferroptosis inhibition improved RGC survival in experimental optic neuropathy models [63]. Similarly, ferroptosis-associated changes were reported in light-induced retinal degeneration, and ferroptosis inhibition attenuated photoreceptor injury [59]. However, reduced GSH levels or altered GPX4 abundance alone do not establish ferroptotic cell death [7,8,9].

#### 2.3.2. Glutathione Peroxidase 4-Independent Pathway

Like the GPX4-dependent pathway, the NAD(P)H/FSP1/coenzyme Q (ubiquinone, CoQ) axis suppresses ferroptosis. Here, NAD(P)H denotes reduced nicotinamide adenine dinucleotide (phosphate). FSP1 is an NAD(P)H-dependent CoQ oxidoreductase that reduces CoQ to CoQH_2_, a lipophilic RTA that limits lipid peroxidation chain reactions, especially at the cell membrane [13]. In non-retinal cancer models of acquired radiotherapy resistance, increased CoQ availability was associated with a shift in reliance from GPX4-dependent to FSP1-dependent antioxidant defense [69]. In ovarian cancer cells, junctional adhesion molecule 3 (JAM3) promoted ferroptosis resistance through nuclear factor erythroid 2-related factor 2 (Nrf2)-dependent upregulation of FSP1 [70]. In the retina, both the NAD(P)H/FSP1/CoQ and GSH/GPX4 defense pathways have been implicated in RPE ferroptosis [71]. Blue light-induced retinal degeneration was associated with lipid peroxidation and disruption of the NAD(P)H/FSP1/CoQ and GSH/GPX4 systems [72]. Because RPE cells provide metabolic and redox support to photoreceptors, impaired RPE antioxidant defenses may indirectly increase photoreceptor vulnerability [57,71]. In RGCs, the GSH/GPX4, NAD(P)H/FSP1/CoQ, and DHODH pathways have been proposed as potential ferroptosis-defense mechanisms [73]; however, the cited review did not provide direct pathway-specific validation of FSP1- and DHODH-dependent protection in retinal neurons.

The GCH1/BH_4_ axis is another GPX4-independent ferroptosis-defense pathway [15,74]. BH_4_ is synthesized de novo from GTP, with GCH1 serving as the rate-limiting enzyme [75]. The GCH1/BH_4_ pathway suppresses lipid peroxidation through antioxidant activity and selective membrane-lipid remodeling [15,75]. Dihydrofolate reductase (DHFR) contributes to the maintenance of the reduced pterin pool by regenerating BH_4_ from its oxidized form, dihydrobiopterin (BH_2_) [75]. In *GCH1*-knockout Jurkat cells, BH_2_ supplementation restored intracellular BH_4_ and reduced sensitivity to the GPX4 inhibitor RAS-selective lethal 3 (RSL3). The DHFR inhibitor methotrexate blocked this rescue. In separate *DHFR*-knockout Karpas-299 experiments, BH_2_ failed to confer protection against RSL3, supporting a requirement for DHFR-dependent BH_4_ regeneration under these conditions [75]. The retinal relevance of BH_4_ is supported by findings that impaired GCH1-dependent BH_4_ synthesis was associated with microglial activation and retinal microvascular degeneration, and that BH_4_ supplementation protected against hyperoxia-induced vascular injury [76]. However, these findings have not established a GCH1/BH_4_-mediated ferroptosis defense in retinal neurons.

DHODH has been reported to reduce CoQ to CoQH_2_ at the mitochondrial inner membrane and suppress mitochondrial lipid peroxidation in selected experimental systems [14,77,78]. Here, CoQ serves as both an electron carrier within the mitochondrial electron transport chain (ETC) and, in its reduced form, CoQH_2_, as an endogenous lipophilic antioxidant [14,77]. Subsequent work challenged the generality of DHODH-mediated ferroptosis protection, showing that a DHODH inhibitor at high concentrations sensitized cells to ferroptosis through off-target FSP1 inhibition rather than DHODH inhibition [64]. In high-glucose-treated ARPE-19 cells, treatment with the Frizzled-7 (FZD7) inhibitor reduced ferroptosis-associated injury and increased DHODH and FSP1 expression without significantly altering GPX4 abundance [79]. These findings suggest potential involvement of GPX4-independent defense mechanisms but do not establish that either DHODH or FSP1 is required for protection.

## 3. Inducers and Inhibitors of Ferroptosis

Chemical compounds and biomolecules capable of inducing (Table 1) or inhibiting (Table 2) ferroptosis continue to be identified and characterized. Ferroptosis inducers act through diverse mechanisms, including impairment of antioxidant defenses and disruption of iron or lipid metabolism [7,8,9,10]. Many of these mechanisms have been established in non-retinal experimental systems; their effects in retinal neurons require cell-type- and model-specific validation. In retinal research, these compounds can serve as experimental probes of lipid-peroxidation-dependent injury, whereas ferroptosis inhibitors are investigated for their potential neuroprotective effects.

### 3.1. Ferroptosis Inducers

Two widely used classes of ferroptosis-inducing compounds (FINs) impair the GSH/GPX4 defense system. Class I FINs, including erastin and sulfasalazine, inhibit system x_c_^−^-mediated cystine uptake, thereby decreasing GSH availability and reducing GPX4-dependent PLOOH detoxification [80,81,82]. Class II FINs include RSL3, ML162, and ML210. RSL3 and ML162 inhibit GPX4, whereas ML210 undergoes intracellular activation to generate a nitrile-oxide electrophile that covalently inhibits GPX4 [83,84,85]. FIN56 promotes ferroptosis through GPX4 degradation and a separate mechanism involving squalene synthase activation and CoQ depletion [86,87,88].

Other compounds affect ferroptosis through context-dependent mechanisms. Early studies linked erastin-induced oxidative non-apoptotic cell death in RAS-transformed cells to interactions with voltage-dependent anion channels 2 and 3 (VDAC2/3) [89]. In glioblastoma cells, temozolomide induced ferroptosis through a DMT1-dependent pathway [90]. ACSL4-associated sorafenib sensitivity has been reported in hepatocellular carcinoma models, although sorafenib does not consistently induce ferroptosis across cancer cells [91,92]. Brequinar was initially reported to promote ferroptosis by disrupting DHODH-associated mitochondrial protection in susceptible cancer models; however, some DHODH inhibitors also inhibited FSP1 at higher concentrations, complicating pathway-specific interpretation [14,64]. Dexamethasone sensitized HT1080 cells to erastin-induced, but not RSL3-induced, ferroptosis through glucocorticoid-receptor-dependent induction of dipeptidase 1 (DPEP1) and GSH depletion [93].

Experimental retinal studies have used erastin and RSL3 to induce injury in ARPE-19 cells and R28 retinal precursor cells [94,95]. Retinal injury triggers, including sodium iodate (NaIO_3_) and excessive all-trans-retinal, have also been reported to engage ferroptotic pathways in photoreceptor-related models [96,97]. However, these injury triggers are not selective ferroptosis inducers, and findings in RPE, retinal precursor, or photoreceptor-derived cell lines should not be assumed to establish equivalent mechanisms in mature photoreceptors or RGCs.

### 3.2. Ferroptosis Inhibitors

Trapping lipid radicals and reducing redox-active iron availability are two established approaches to inhibit ferroptosis [7,62,80]. Fer-1, liproxstatin-1, and α-tocopherol (a form of vitamin E) inhibit lipid-peroxidation propagation through radical-trapping activity, whereas deferoxamine reduces iron-dependent oxidative damage by chelating iron [7,62,80]. N-acetylcysteine (NAC) can replenish intracellular cysteine and support GPX4-dependent protection. Recent work also demonstrated that NAC and cysteine could act as alternative reducing substrates for GPX4, particularly under GSH-deficient conditions, indicating that their protection need not depend exclusively on GSH replenishment [98]. β-mercaptoethanol (β-ME) facilitated cysteine availability through mixed-disulfide formation and could bypass the requirement for system x_c_^−^-mediated cystine uptake in experimental ferroptosis models [81].

Experiments in retinal injury models support the protective potential of these agents. Fer-1 attenuated light-induced photoreceptor degeneration [59], improved RGC survival in optic nerve crush (ONC) and microbead-induced glaucoma models [63], and reduced pathological angiogenesis in OIR [60]. In retinal ischemia/reperfusion (I/R) injury, deferoxamine protected retinal structure and function [99], while liproxstatin-1 preserved retinal structure and partially restored electroretinographic responses [100]. Primaquine also attenuated erastin- and RSL3-induced injury in R28 cells, and glutathione S-transferase alpha 1 (*Gsta1*) knockdown provided mechanistic evidence linking GSTA1 to primaquine-mediated protection [95]. In photoreceptor-derived 661W cells, NAC and exogenous GSH reduced NaIO_3_-associated injury [96], while the 5-LOX inhibitor zileuton reduced ferroptosis-associated injury in RPE experiments [94].

Together, these findings support the protective potential of interventions targeting iron-dependent lipid peroxidation across several experimental systems. However, these benefits may also involve broader antioxidant or pathway-modulating mechanisms. Pharmacological rescue alone does not establish that protection is mediated specifically by ferroptosis inhibition [7,8,9].

## 4. Ferroptosis-Associated Injury in Retinal and Optic Nerve Diseases

**Figure 4:**
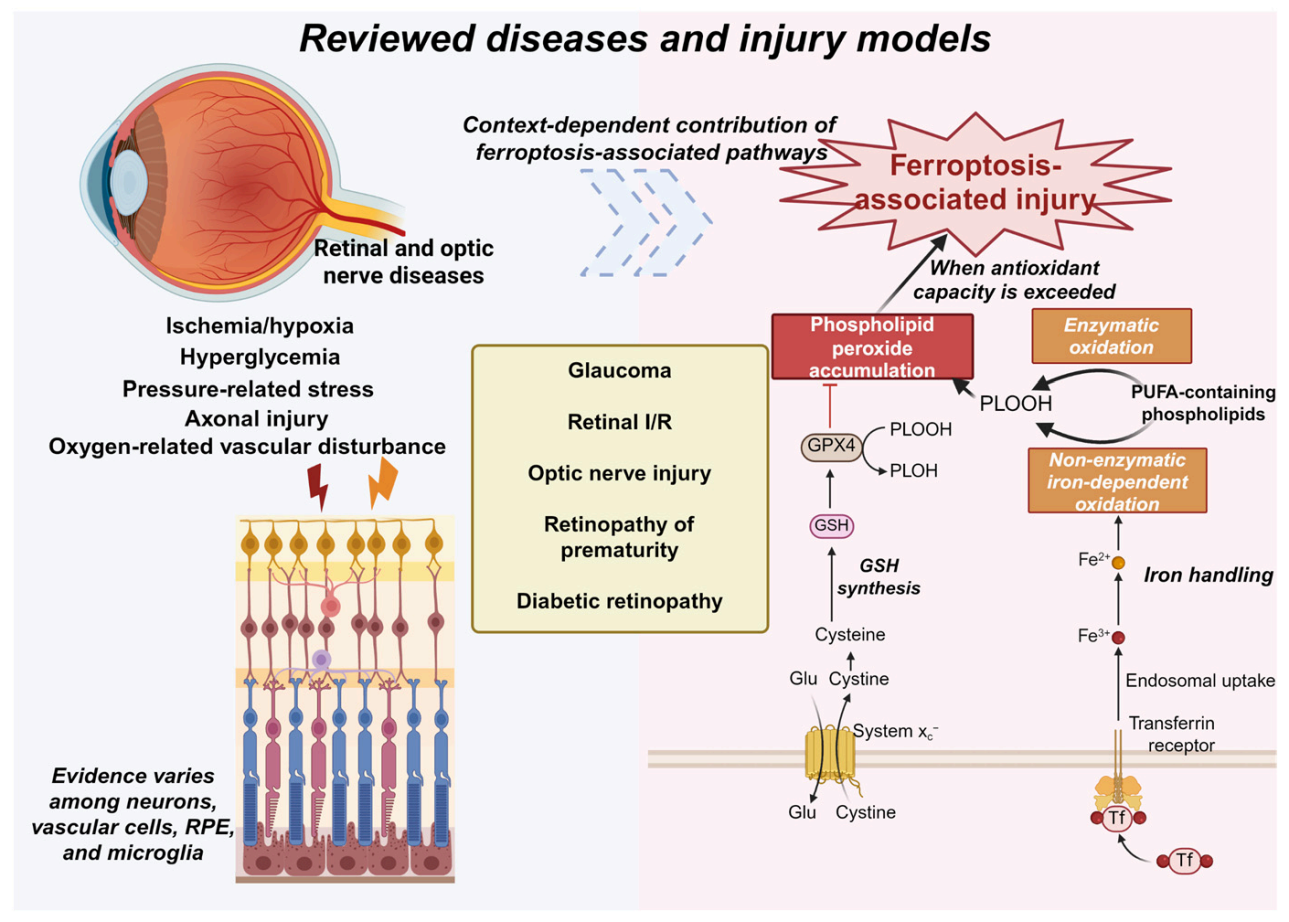
Overview of ferroptosis-associated pathways in retinal and optic nerve diseases. The diseases and experimental models discussed in this review involve distinct initiating stresses, including ischemia/hypoxia, hyperglycemia, pressure-related stress, axonal injury, and oxygen-related vascular disturbance. Experimental evidence implicates changes in iron handling, phospholipid oxidation, and antioxidant defenses in a model- and cell-type-dependent manner. Peroxide accumulation can contribute to ferroptosis-associated injury when antioxidant capacity is exceeded. The dashed arrows denote context-dependent experimental evidence rather than a universally established or exclusive mechanism in every listed disorder. The lightning-bolt symbols indicate oxidative injury. Abbreviations: Fe^2+^, ferrous iron; Fe^3+^, ferric iron; Glu, glutamate; GPX4, glutathione peroxidase 4; GSH, reduced glutathione; I/R, ischemia/reperfusion; PLOH, phospholipid alcohol; PLOOH, phospholipid hydroperoxide; PUFA, polyunsaturated fatty acid; RPE, retinal pigment epithelium; Tf, transferrin; system x_c_^−^, cystine-glutamate antiporter. Created in BioRender. Xiang, L. (2026) https://BioRender.com/c4xowuc.

Ferroptosis-related mechanisms, including disturbances in iron handling, lipid peroxidation, and antioxidant defenses, have been implicated in neuronal and non-neuronal retinal injury (Figure 4) [4,61,63]. However, the strength and nature of the evidence vary across disease models and retinal cell types. Accordingly, the following sections discuss ferroptosis-associated changes, considering the experimental models, cell types, pharmacological rescue, and mechanistic findings in each study.

### 4.1. Ferroptosis and Diabetic Retinopathy

Diabetic retinopathy (DR) is a complication of diabetes mellitus involving the retinal neurovascular unit (NVU), with abnormalities in cellular interactions, retinal vascular integrity, and blood-retinal barrier (BRB) function [101]. DR is broadly classified as non-proliferative diabetic retinopathy (NPDR) and PDR [101]. As DR advances, retinal lesions develop, including microaneurysms, hard exudates, cotton-wool spots, pathological neovascularization, vitreoretinal proliferation, macular edema, and, in severe stages, potential retinal detachment [101]. Existing interventions include laser photocoagulation (focal and panretinal), intravitreal injections of anti-VEGF (vascular endothelial growth factor) agents or corticosteroids, and vitrectomy [101,102], depending on the clinical indication. Nevertheless, these interventions do not address all mechanisms of retinal injury. Chronic hyperglycemia promotes oxidative stress and disrupts retinal cellular homeostasis, providing a biological context for investigating ferroptotic cell death. However, these changes alone do not establish ferroptotic cell death [7,9,103,104].

#### 4.1.1. Evidence from Whole-Retina and Neuronal Models

Experimental diabetic-retina studies have reported increased iron and lipid-peroxidation indices, reduced GSH, and altered expression of iron-handling and antioxidant proteins [105,106]. In a rat model of DR, Shao et al. observed increased oxidative stress markers in retinal tissue and peripheral blood, together with reduced retinal GPX4 expression and an altered GSH/glutathione disulfide (GSSG) balance. Fer-1 administration improved antioxidant indices and reduced retinal tissue injury, consistent with involvement of ferroptosis-associated processes [106]. However, Fer-1 also reduced apoptosis- and pyroptosis-associated indices; these findings do not isolate ferroptotic cell death as the mechanism responsible for retinal protection.

Likewise, in a streptozotocin (STZ)-induced mouse model of DR, pharmacological fatty acid-binding protein 4 (FABP4) inhibition was shown to confer retinal protection by limiting lipid peroxidation and oxidative stress in retinal tissues through increased peroxisome proliferator-activated receptor γ (PPARγ) expression [107]. This intervention provides evidence that an upstream metabolic regulator influences retinal injury. Nevertheless, protection following FABP4 suppression should not be equated with specific validation of ferroptotic cell death, because the intervention may also alter lipid metabolism and broader oxidative stress.

More direct neuronal evidence came from experiments implicating ferroptotic cell death in photoreceptor degeneration in diabetic mice and high-glucose-treated photoreceptor-derived 661W cells. Increased iron and MDA, reduced GSH, and changes in GPX4, SLC7A11, ACSL4, ferritin heavy chain 1 (FTH1), and nuclear receptor coactivator 4 (NCOA4) were accompanied by photoreceptor mitochondrial abnormalities. Fer-1 improved cell viability *in vitro* and attenuated biochemical, protein-expression, and mitochondrial abnormalities in the tested models [105]. Compared with whole-retina analyses, photoreceptor-specific localization of protein changes and mitochondrial injury provided stronger evidence of neuronal involvement. Nevertheless, the evidence relies mainly on biochemical, morphological, and pharmacological findings without cell-type-specific genetic validation. Moreover, 661W cells do not fully reproduce mature photoreceptor physiology. These results support involvement of ferroptosis-associated processes in diabetic photoreceptor injury but do not establish ferroptotic cell death as the exclusive injury mechanism.

#### 4.1.2. Direct Retinal Endothelial Evidence

Pathologically, BRB dysfunction contributes to vascular leakage and edema in DR [101,108]. Anatomically, the BRB comprises the inner blood–retinal barrier (iBRB) and outer blood–retinal barrier (oBRB) [108]. The iBRB consists mainly of continuous retinal microvascular endothelial cells connected by tight junctions, whereas the oBRB consists of RPE cells interconnected by tight junctions, creating a barrier that separates the fenestrated choriocapillaris from the overlying neural retina [108].

Among the retinal endothelial studies, Zhang et al. provided pathway-specific evidence using human retinal capillary endothelial cells (HRCECs) under hyperglycemic conditions. High glucose inhibited cell growth, and Fer-1 reversed this effect. Tripartite motif-containing protein 46 (*TRIM46*) overexpression increased susceptibility to injury, whereas *TRIM46* knockdown was protective. The investigators further showed that TRIM46 interacted with GPX4 and promoted its ubiquitination and degradation; GPX4 overexpression counteracted TRIM46-associated injury, while RSL3 reversed the protection afforded by *TRIM46* silencing [109]. These experiments provide mechanistic support for a TRIM46-GPX4 pathway in high-glucose-exposed HRCECs. Nevertheless, the contribution of this pathway to barrier dysfunction *in vivo* and vascular leakage in patients has not been established.

Other retinal endothelial studies implicated regulatory RNAs in ferroptosis-associated changes. Liu et al. examined the ZFAS1/miR-7-5p/ACSL4 axis in human retinal endothelial cells (hRECs), linking regulation of ACSL4 to high-glucose-associated injury [110]. Zhan et al. reported that 25-hydroxyvitamin D3 reduced oxidative stress and ferroptosis-associated changes in high-glucose-treated human retinal microvascular endothelial cells (HRMECs) through a miR-93-associated mechanism [111]. These studies provide evidence for regulatory involvement of the proposed RNA pathways, but the specificity of the associated cell-death mechanisms remains less firmly established.

#### 4.1.3. Complementary Non-Retinal Endothelial Evidence

Non-retinal endothelial experiments provided mechanistic context but should be considered separately from direct ocular evidence. Luo et al. investigated high-glucose- and interleukin-1β (IL-1β)-associated injury in human umbilical vein endothelial cells (HUVECs), implicating the p53/SLC7A11/GSH pathway and reporting associated GPX4 changes [112]. Xiao et al. investigated erastin-treated HUVECs and found that miR-17-92 overexpression suppressed ferroptosis-associated changes through the A20/ACSL4 axis [113]. Although these findings suggest regulatory mechanisms potentially relevant to endothelial ferroptotic cell death, it should be noted that HUVECs differ from retinal microvascular endothelial cells, and erastin exposure does not reproduce the diabetic retinal environment. These studies therefore do not directly demonstrate protection of the diabetic retinal vasculature.

#### 4.1.4. Retinal Pigment Epithelium Mechanisms and Their Implications for Neuronal Support

RPE studies provided additional ocular evidence, although RPE injury should not be presented as direct neuronal ferroptotic cell death. Liu et al. reported that extracellular glia maturation factor-β (GMFB) displaced ATP6V1A from lysosomes in RPE cells, impairing lysosomal acidification and chaperone-mediated autophagic degradation of ACSL4 [114]. Treatment with a GMFB antibody, a lysosomal activator, a chaperone-mediated autophagy activator, or liproxstatin-1 provided complementary support for this mechanism, with protective effects also observed in experimental early DR [114]. This combination of mechanistic investigation and rescue at different points in the pathway provides stronger support than changes in oxidative stress markers alone. However, the broader biological effects of these interventions limit attribution of the observed retinal protection solely to inhibition of RPE ferroptotic cell death.

Additional studies have investigated thioredoxin-interacting protein (TXNIP)-associated mitochondrial dysfunction, ferritinophagy, and ferroptosis-associated changes in high-glucose-exposed RPE cells [115]; however, without direct pathway-specific experiments, the complete causal sequence should not be assumed. In high-glucose-treated ARPE-19 cells, *circ-PSEN1* knockdown reduced ferroptosis-associated changes through the miR-200b-3p/cofilin-2 axis, while inhibition of miR-200b-3p partially reversed these effects [116]. Astragaloside-IV-associated protection has also been linked to the miR-138-5p/Sirtuin 1 (Sirt1)/Nrf2 pathway in ARPE-19 cells [117]. These findings suggest potential mechanisms of RPE injury, with possible implications for outer-retinal support and oBRB function. However, direct links between the individual molecular pathways described above and diabetic macular edema require further validation, although BRB dysfunction is an established contributor to diabetic macular edema [118].

#### 4.1.5. Remaining Limitations

Increased ROS, MDA, or iron levels, reduced GPX4 abundance, and altered GSH levels do not individually establish ferroptotic cell death. Stronger attribution requires converging evidence from cell-death assessments, membrane lipid-peroxidation measurements, tests of iron dependence, appropriate rescue experiments, and, where feasible, genetic validation. A further limitation is that high-glucose-treated cultured cells and diabetic animal models do not necessarily isolate ischemia as the initiating stimulus; hyperglycemic and ischemic injury should therefore not be treated as interchangeable.

Collectively, these findings identify ferroptosis-associated pathways as potential therapeutic targets in experimental DR, but their cell-type-specific contributions and clinical relevance require further validation.

### 4.2. Ferroptosis and Glaucoma

Glaucoma is a major cause of irreversible blindness worldwide and is characterized by progressive degeneration of RGCs and their axons [119,120]. Elevated intraocular pressure (IOP) is the principal modifiable risk factor, but glaucomatous degeneration can also occur within the statistically normal IOP range, as in normal-tension glaucoma (NTG), highlighting the importance of additional susceptibility factors [119,120]. Because IOP-lowering treatment does not halt RGC loss in all patients [121], neuroprotective strategies that preserve RGC survival and visual function may complement current therapy [122].

Growing preclinical evidence suggests that ferroptotic cell death may be involved in glaucoma [73]. Epidemiological studies have reported associations between glaucoma and iron supplementation [123] or elevated serum Ft [124]. These observations support further study of iron metabolism but do not establish retinal iron overload or ferroptotic cell death as a cause of disease. In glutamate-aspartate transporter (GLAST) knockout mice, a model of NTG, Fer-1 reduced RGC loss and preserved retinal structure, supporting a contribution of lipid-peroxidation-associated injury [125]. Oral deferiprone, an iron-chelating agent, improved RGC survival and attenuated optic nerve degeneration in a magnetic microbead-induced ocular hypertension model [126]. In an acute pathologically high intraocular pressure (ph-IOP) model, NCOA4-mediated degradation of FTH1 contributed to retinal iron accumulation. *In vivo*, *Ncoa4* knockdown reduced FTH1 degradation and Fe^2+^ accumulation, and deferiprone protected RGCs and visual function [127]. While these complementary findings strengthen the link between iron regulation and RGC injury, the specificity of the death mechanism requires consideration of the accompanying lipid peroxidation and rescue evidence.

In an N-methyl-D-aspartate (NMDA)-induced retinal excitotoxicity model, intravitreal NMDA was associated with Fe^2+^ buildup in the rat retina, and zinc-deferoxamine treatment reduced Fe^2+^ accumulation and mitigated lipid peroxidation, consistent with ferroptosis-associated changes [128]. A study combining NMDA-induced retinal injury *in vivo* with glutamate-treated R28 cells *in vitro* linked excitotoxic injury to increased p38 mitogen-activated protein kinase (MAPK) phosphorylation, altered spermidine/spermine N^1^-acetyltransferase 1 (SAT1) expression, and reduced FTL, SLC7A11, and GPX4 expression. Conversely, the p38 inhibitor SB202190 protected RGCs and reversed these ferroptosis-associated changes in the tested models [129]. Since p38 regulates multiple injury pathways, this protection alone does not establish that the benefit results specifically from inhibition of ferroptotic cell death. In an NMDA-induced retinal injury model, bone morphogenetic protein 4 (BMP4)-GPX4 signaling was associated with reduced oxidative stress and iron accumulation and improved retinal stem-cell transplantation outcomes, including enhanced survival and differentiation [130]. Moreover, *Dexras1* deletion reduced NMDA-induced iron uptake and neurotoxicity in primary cortical cultures and protected RGCs after intravitreal NMDA in mice [131]. These findings support a role for Dexras1-dependent iron uptake in excitotoxic injury, consistent with the previously described Dexras1/peripheral-type benzodiazepine receptor-associated protein 7 (PAP7)/DMT1 pathway [132]. However, the complete pathway has not been directly validated in RGCs, and the study does not specifically establish ferroptotic cell death.

Complementary studies in aqueous outflow addressed mechanisms that may influence IOP rather than directly demonstrating ferroptotic cell death in retinal neurons. Dysfunction of the trabecular meshwork (TM) and Schlemm’s canal (SC) can increase resistance to aqueous-humor outflow and contribute to IOP elevation [133]. Mechanical stretching induced ferroptosis-associated changes in human trabecular meshwork cells (HTMCs), which were attenuated by *Piezo1* (a mechanically activated ion channel protein) knockdown. Pharmacological Piezo1 activation with Yoda1 produced similar responses, and suppression of the c-Jun N-terminal kinase (JNK)/p38 signaling pathway reduced these effects [134]. In dexamethasone-associated models, transmembrane and coiled-coil domain protein 1 (TMCO1) upregulation promoted extracellular matrix (ECM) deposition and ferroptosis-associated changes in HTMCs, with reduced extracellular signal-regulated kinase (ERK) phosphorylation implicated in the mechanism [135]. These TM findings are relevant to outflow pathology but should be kept distinct from evidence of RGC ferroptotic cell death.

Acute IOP elevation followed by reperfusion is widely used to model retinal I/R injury relevant to glaucoma, although it does not reproduce all features of chronic glaucomatous degeneration [136]. Single-cell RNA sequencing identified ferroptosis-associated transcriptional changes after retinal I/R injury, and treatment targeting ferroptosis attenuated inflammatory and immune responses and improved RGC survival [137]. Vitamin K1 treatment reduced retinal inflammation and microglial ferroptosis-associated changes in an acute ocular-hypertension model [138]. The reported mechanism centers on microglial responses and should be distinguished from direct evidence of neuron-autonomous protection. *Sox9* knockdown reduced oxidative stress, inflammatory responses, lipid ROS, and ferroptosis-associated biochemical changes in 661W cells subjected to oxygen-glucose-deprivation/reoxygenation. An ERK-pathway agonist partially reversed the protection [139]. Although the study also examined retinal I/R injury *in vivo*, the cultured-cell findings should not be presented as RGC-specific evidence.

Deferoxamine preserved retinal morphology and visual function after I/R, accompanied by altered Tf expression and enhanced GPX4-associated antioxidant defense [99]. These findings support a role for iron-dependent oxidative injury but do not independently establish ferroptotic cell death as the specific mechanism. NCOA4, also known as androgen receptor-associated protein of 70 kDa (ARA70), is the cargo receptor for ferritinophagy. Its expression increased in RGCs after retinal I/R, promoting ferritinophagy and increasing cellular Fe^2+^ concentration [140], consistent with the NCOA4-FTH1 mechanism described in the ph-IOP model [127]. Additionally, tetrahedral framework nucleic acid-mediated delivery of DJ-1 (protein deglycase)-targeting small activating RNA (saRNA) protected against retinal I/R injury and was associated with upregulation of the SLC7A11/GPX4 axis [141].

Taken together, these studies provide model-dependent evidence linking altered iron handling, lipid peroxidation, and impaired antioxidant defenses to RGC injury. The combination of genetic perturbation and pharmacological protection offers stronger mechanistic support in some models than marker changes alone. However, chronic experimental glaucoma, acute high-IOP retinal injury, and NMDA-induced excitotoxicity represent distinct experimental settings and should not be treated as interchangeable. Future studies should determine whether cell-type-specific manipulation of ferroptosis-associated pathways preserves RGCs, optic nerve axons, and relevant functional outcomes during chronic glaucomatous degeneration.

### 4.3. Ferroptosis and Retinopathy of Prematurity

ROP is a disorder of retinal vascular development in premature infants [142]. Early disruption of physiological vascular growth can produce an avascular retina, followed by hypoxia-driven pathological neovascularization as retinal metabolic demand increases. Severe ROP can cause lasting visual impairment or blindness in children worldwide. Experimental OIR reproduces selected vascular features of ROP but does not capture the complete clinical disease [143]. Niu et al. investigated endothelial injury associated with C-X-C motif chemokine ligand 10 (CXCL10)/C-X-C motif chemokine receptor 3 (CXCR3) using H_2_O_2_-damaged HRMECs and OIR-subjected mice. Curcumin treatment reduced ferroptosis-associated changes, preserved retinal vascular structures during hyperoxic exposure, and reduced subsequent pathological neovascularization [144]. However, curcumin’s broad antioxidant and anti-inflammatory effects limit attribution of its protective effects specifically to inhibition of ferroptotic cell death. Zhou et al. examined heme oxygenase 1 (HO-1) in OIR-subjected mice and HRMECs. *HO-1* knockdown increased GPX4 abundance and reduced lipid peroxidation, while HO-1 inhibition reduced pathological neovascularization *in vivo* and endothelial migration and tube formation *in vitro* [145]. However, these findings do not independently confirm whether ferroptotic cell death accounts for all the observed vascular damage. Meanwhile, Fer-1 administration markedly reduced pathological neovascularization and avascular areas in OIR, providing complementary pharmacological evidence [60]. Together, these studies suggest that ferroptosis-associated pathways may contribute to vascular injury and remodeling in OIR. However, vascular preservation, pathological neovascularization, and endothelial death are distinct outcomes, and their causal relationships require further clarification. The relevance of these findings to clinical ROP and direct retinal neuronal injury remains to be established.

### 4.4. Ferroptosis and Traumatic Optic Neuropathy

Traumatic optic neuropathy (TON) results from direct or indirect injury to the optic nerve following ocular or craniofacial trauma [146]. Mechanical injury, vascular compromise, and secondary optic nerve swelling within the confined optic canal may contribute to axonal injury and subsequent RGC loss [146]. Concurrent central retinal artery occlusion has been reported after blunt trauma [147], but retinal ischemia (RI) is not a defining feature of all TON [146]. RGCs are specialized neurons in the retina whose axons bundle together to form the optic nerve, transmitting visual signals from the eye to the brain [148]. Experimental ONC damages these axons and induces subsequent RGC degeneration, providing a model for investigating selected mechanisms of traumatic optic nerve injury [149].

Guo et al. reported reduced GPX4 expression, increased iron and lipid peroxides, and mitochondrial abnormalities after ONC in rats. In this model, Fer-1 improved RGC survival, preserved retinal nerve fiber layer thickness, and improved flash visual evoked potential responses. In mice with microbead-induced ocular hypertension, Fer-1 preserved RGCs without reducing IOP. Fer-1 also reduced mitochondrial lipid-peroxide accumulation, preserved mitochondrial ATP production, and prevented downregulation of selected mitochondrial genes (*mt-Cytb* and *mt-Atp6*) [63].

Lin et al. investigated deferoxamine nanoparticles in an experimental TON model. Treatment protected against RGC death, reduced cyclooxygenase-2 (COX-2) expression, and prevented GPX4 inactivation. Increased COX-2 expression and lipid-peroxide accumulation after injury were consistent with ferroptosis-associated changes [150]. However, the protection observed with iron chelation does not distinguish ferroptotic cell death from broader iron-dependent oxidative injury.

## 5. Conclusions and Future Directions

Current evidence supports a potential, context-dependent contribution of ferroptosis-associated pathways to retinal and optic nerve injury in experimental models relevant to DR, glaucoma, retinal I/R injury, ROP, and TON. Comparing these models highlights the need to distinguish neuronal injury, non-neuronal dysfunction, and vascular remodeling, and to interpret the evidence according to the outcome assessed. Mechanistic support is stronger when complementary functional and molecular findings converge than when conclusions rely on isolated marker changes or a single rescue intervention. This review complements existing pathway-focused discussions by identifying where the evidence supports cell-type-specific mechanisms and where interpretation remains provisional.

Three unresolved questions could guide further investigation. First, which retinal cell types undergo ferroptotic cell death, and which pathway changes are causal rather than secondary to injury? Studies should combine cell-type-specific measurements of cell death, assessments of membrane lipid peroxidation, and tests of iron dependence with appropriate pharmacological rescue experiments and, where feasible, genetic perturbation. Neuron-autonomous protection should be distinguished from indirect neuronal protection mediated by effects on RPE, vascular endothelial, or glial cells. Studies should determine whether protection of these non-neuronal cells preserves neuronal structure and function.

Second, to what extent do mechanisms identified in acute injury models contribute to chronic disease progression? Existing experimental evidence suggests that ferroptosis-associated injury is not restricted to acute insults, with findings also reported in models of glaucoma and diabetic retinal degeneration [63,105,125]. However, it remains unclear whether these cell-type-specific mechanisms contribute causally during chronic disease progression and whether targeting them provides sustained protection after degeneration is established.

Third, does targeting these pathways preserve visual function? Future studies should assess sustained functional outcomes alongside neuronal survival and, where appropriate, optic nerve axon preservation in disease-relevant models. Evaluation of retinal and visual-pathway function should be integrated with retinal structural assessments and cell-type-specific mechanistic evidence.

Before considering clinical translation, priority should therefore be given to reproducible evidence that ferroptosis-associated mechanisms causally contribute to retinal injury and that targeting these mechanisms after disease onset provides sustained structural and functional protection in disease-relevant experimental models. This evidence should be complemented by demonstration of adequate ocular exposure, effective target engagement, and acceptable ocular and systemic safety.

Meeting these requirements depends on addressing practical obstacles, including limitations in ocular drug delivery, compound-specific toxicity, and the lack of validated clinical biomarkers. In particular, the eye’s unique anatomy and physiology can hinder the maintenance of therapeutic drug concentrations at the target site. The presence of ocular delivery barriers, including the anterior segment barriers (e.g., blinking, tear turnover, and nasolacrimal drainage) and posterior segment barriers (e.g., the BRB), limits drug bioavailability. In addition, systemic iron chelation can cause agent-, dose-, and exposure-dependent adverse effects. For example, the drug label for deferoxamine describes ocular and auditory toxicity, particularly with prolonged treatment, higher doses, and low Ft levels. It also warns of an increased risk of *Yersinia* infections and reports mucormycosis, including fatal cases [151]. These risks underscore the need for an appropriate dosing regimen. However, achieving therapeutic drug concentrations in the eye through systemic administration without causing toxicity remains a substantial translational challenge. Direct mechanistic validation in living human retinal tissue is constrained by the risks of invasive sampling and limited opportunities to obtain tissue, highlighting the need for validated non-invasive biomarkers of target engagement and treatment response. Such biomarkers would complement, rather than replace, the evidence of preserved visual function.

Taken together, ferroptosis-associated pathways remain potential experimental targets rather than established treatment strategies for retinal and optic nerve diseases. Progress will depend on linking cell-type-specific mechanisms to neuronal protection in disease-relevant models while establishing feasible delivery methods and acceptable safety profiles. These priorities provide a framework for assessing whether experimental retinal protection can translate into meaningful preservation of vision.

## Figures and Tables

**Table 1:** Representative ferroptosis inducers, sensitizers, and retinal injury triggers.

Category		Compound or Trigger	Mechanism or Reported Effect
Antioxidant System	Cystine uptake	Erastin	Inhibits system x_c_^−^, reducing cystine uptake and GSH-dependent protection
		Sulfasalazine	Inhibits system x_c_^−^; ferroptosis induction is context dependent
	GPX4 inhibition	RSL3	Inhibits GPX4 and promotes phospholipid-hydroperoxide accumulation
		ML162	Covalently inhibits GPX4
		ML210	Undergoes intracellular activation to generate a nitrile-oxide electrophile that covalently inhibits GPX4
	GPX4/CoQ depletion	FIN56	Promotes GPX4 degradation and CoQ depletion
	DHODH inhibition	Brequinar	Compromises mitochondrial defense; off-target FSP1 inhibition may contribute at higher concentrations
	GSH depletion	Dexamethasone	Induces DPEP1 and GSH depletion, sensitizing HT1080 cells to erastin-induced ferroptosis
Iron metabolism	Iron handling	Temozolomide	Promotes DMT1-dependent ferroptosis in glioblastoma cells
Lipid metabolism	Context-dependent action	Sorafenib	ACSL4-associated sensitivity in selected cancer models; not a consistent ferroptosis inducer
Retinal-injury triggers	Oxidative injury	Sodium iodate	Induces ferroptosis-associated retinal-cell injury, including GSH depletion and lipid peroxidation
	Retinaldehyde stress	Excess all-trans-retinal	Engages ferroptotic injury in photoreceptor-related models

Table legend: Canonical probes, sensitizers, and retinal injury triggers are distinguished because they differ in specificity. Sodium iodate and excess all-trans-retinal are not selective ferroptosis inducers. Evidence from cancer cells or retinal cell lines does not necessarily establish the same mechanism in mature retinal neurons. Abbreviations: ACSL4, acyl-CoA synthetase long-chain family member 4; CoQ, coenzyme Q (ubiquinone); DHODH, dihydroorotate dehydrogenase; DMT1, divalent metal transporter 1; DPEP1, dipeptidase 1; FIN, ferroptosis-inducing compound; FSP1, ferroptosis suppressor protein 1; GPX4, glutathione peroxidase 4; GSH, reduced glutathione; RSL3, RAS-selective lethal 3; system x_c_^−^, cystine-glutamate antiporter.

**Table 2:** Representative ferroptosis inhibitors and retinal protective agents.

Category		Compound	Mechanism or Reported Effect
Lipophilic RTAs	Radical trapping	Ferrostatin-1	Limits lipid-radical propagation; protection reported in photoreceptor, RGC, and OIR models
		Liproxstatin-1	Limits lipid-peroxidation propagation; investigated in retinal I/R
		α-Tocopherol	Traps lipid peroxyl radicals
Iron chelators	Iron chelation	Deferoxamine	Reduces redox-active iron availability; protection reported in retinal I/R
		Deferiprone	Chelates iron; preserves RGCs in experimental glaucoma
Antioxidant axis activators	GPX4 support	NAC	Supports cysteine availability and can serve as a GPX4-reducing substrate
	Redox support	Exogenous GSH	Reduces lipid peroxidation and injury in sodium-iodate-treated 661W cells
	Cysteine supply	β-mercaptoethanol	Facilitates cysteine acquisition and bypasses system x_c_^−^ dependence
Others	5-LOX inhibition	Zileuton	Reduces ferroptosis-associated RPE injury
	GSTA1 modulation	Primaquine	GSTA1-dependent protection reported in R28 cells

Table legend: These agents may also affect oxidative stress or other injury pathways. Pharmacological protection alone does not establish ferroptosis specificity; interpretation requires complementary biochemical and, where feasible, genetic evidence. Abbreviations: GPX4, glutathione peroxidase 4; GSH, reduced glutathione; GSTA1, glutathione S-transferase alpha 1; I/R, ischemia/reperfusion; LOX, lipoxygenase; NAC, N-acetylcysteine; OIR, oxygen-induced retinopathy; RGC, retinal ganglion cell; RPE, retinal pigment epithelium; RTA, radical-trapping antioxidant.

## Data Availability

No new data were created or analyzed in this study. Data sharing is not applicable to this article.

## Abbreviations

ACSL4	acyl-CoA synthetase long-chain family member 4
AKT	protein kinase B
ARA70	androgen receptor-associated protein of 70 kDa
BH_2_	dihydrobiopterin
BH_4_	tetrahydrobiopterin
BMP4	bone morphogenetic protein 4
BRB	blood-retinal barrier
CoQ	coenzyme Q (ubiquinone)
CoQH_2_	reduced coenzyme Q (ubiquinol)
COX-2	cyclooxygenase-2
Cp	ceruloplasmin
CXCL10	C-X-C motif chemokine ligand 10
CXCR3	C-X-C motif chemokine receptor 3
CYB5R1	cytochrome b5 reductase 1
DHA	docosahexaenoic acid
DHFR	dihydrofolate reductase
DHODH	dihydroorotate dehydrogenase
DJ-1	protein deglycase
DMT1	divalent metal transporter 1
DPEP1	dipeptidase 1
DR	diabetic retinopathy
ECM	extracellular matrix
ERK	extracellular signal-regulated kinase
ETC	electron transport chain
FABP4	fatty acid-binding protein 4
Fe2+	ferrous iron
Fe3+	ferric iron
Fer-1	ferrostatin-1
FIN	ferroptosis-inducing compound
Fpn	ferroportin
FSP1	ferroptosis suppressor protein 1
Ft	ferritin
FTH	ferritin heavy chain
FTH1	ferritin heavy chain 1
FTL	ferritin light chain
FZD7	Frizzled-7
GCH1	GTP cyclohydrolase 1
GCL	ganglion cell layer
GLAST	glutamate-aspartate transporter
Glu	glutamate
GMFB	glia maturation factor-β
GPX4	glutathione peroxidase 4
GSH	reduced glutathione
GSSG	glutathione disulfide
GSTA1	glutathione S-transferase alpha 1
GTP	guanosine triphosphate
H2O2	hydrogen peroxide
Hepc	hepcidin
Heph	hephaestin
HO-1	heme oxygenase 1
HRCEC	human retinal capillary endothelial cell
hREC	human retinal endothelial cell
HRMEC	human retinal microvascular endothelial cell
HTMC	human trabecular meshwork cell
HUVEC	human umbilical vein endothelial cell
iBRB	inner blood–retinal barrier
IL-1β	interleukin-1β
IOP	intraocular pressure
I/R	ischemia/reperfusion
JAM3	junctional adhesion molecule 3
JNK	c-Jun N-terminal kinase
LC-PUFA	long-chain polyunsaturated fatty acid
LIP	labile iron pool
LOX	lipoxygenase
LPCAT3	lysophosphatidylcholine acyltransferase 3
LPS	lipopolysaccharide
MAPK	mitogen-activated protein kinase
MBOAT1/2	membrane-bound O-acyltransferase domain-containing 1 and 2
MDA	malondialdehyde
mTORC1	mechanistic target of rapamycin complex 1
MUFA	monounsaturated fatty acid
NAC	N-acetylcysteine
NAD(P)^+^	oxidized nicotinamide adenine dinucleotide (phosphate)
NAD(P)H	reduced nicotinamide adenine dinucleotide (phosphate)
NaIO_3_	sodium iodate
NCOA4	nuclear receptor coactivator 4
NMDA	N-methyl-D-aspartate
NPDR	non-proliferative diabetic retinopathy
Nrf2	nuclear factor erythroid 2-related factor 2
NTBI	non-transferrin-bound iron
NTG	normal-tension glaucoma
NVU	neurovascular unit
oBRB	outer blood–retinal barrier
OIR	oxygen-induced retinopathy
ONC	optic nerve crush
PAP7	peripheral-type benzodiazepine receptor-associated protein 7
PDR	proliferative diabetic retinopathy
ph-IOP	pathologically high intraocular pressure
PI3K	phosphoinositide 3-kinase
PL	phospholipid
PLOH	phospholipid alcohol
PLOOH	phospholipid hydroperoxide
POR	cytochrome P450 oxidoreductase
PPARα	peroxisome proliferator-activated receptor α
PPARγ	peroxisome proliferator-activated receptor γ
PUFA	polyunsaturated fatty acid
PUFA-CoA	polyunsaturated fatty acyl-coenzyme A
PUFA-PL	polyunsaturated fatty acid-containing phospholipid
RCD	regulated cell death
RGC	retinal ganglion cell
RI	retinal ischemia
ROP	retinopathy of prematurity
ROS	reactive oxygen species
RPE	retinal pigment epithelium
RSL3	RAS-selective lethal 3
RTA	radical-trapping antioxidant
saRNA	small activating RNA
SAT1	spermidine/spermine N^1^-acetyltransferase 1
SC	Schlemm’s canal
SCD1	stearoyl-CoA desaturase-1
Sirt1	sirtuin 1
SLC7A11	solute carrier family 7 member 11
SREBP1	sterol regulatory element-binding protein 1
STEAP3	six-transmembrane epithelial antigen of the prostate 3
STZ	streptozotocin
system x_c_^-^	cystine-glutamate antiporter
TBI	transferrin-bound iron
Tf	transferrin
TfR	transferrin receptor
TfR1	transferrin receptor 1
TfR2	transferrin receptor 2
TLR4	Toll-like receptor 4
TM	trabecular meshwork
TMCO1	transmembrane and coiled-coil domain protein 1
TON	traumatic optic neuropathy
TRIM46	tripartite motif-containing protein 46
TXNIP	thioredoxin-interacting protein
VEGF	vascular endothelial growth factor
VDAC2/3	voltage-dependent anion channels 2 and 3
ZIP8	Zrt- and Irt-like protein 8
ZIP14	Zrt- and Irt-like protein 14
β-ME	β-mercaptoethanol
•OH	hydroxyl radical
